# The Effect of Integration of Self-Management Web Platforms on Health Status in Chronic Obstructive Pulmonary Disease Management in Primary Care (e-Vita Study): Interrupted Time Series Design

**DOI:** 10.2196/jmir.8262

**Published:** 2017-08-16

**Authors:** Esther PWA Talboom-Kamp, Noortje A Verdijk, Marise J Kasteleyn, Lara M Harmans, Irvin JSH Talboom, Ingrid Looijmans-van den Akker, Nan van Geloven, Mattijs E Numans, Niels H Chavannes

**Affiliations:** ^1^ Leiden University Medical Center Public Health and Primary Care Department Leiden Netherlands; ^2^ Saltro Diagnostic Center Utrecht Netherlands; ^3^ Zorgdraad Foundation Oosterbeek Netherlands; ^4^ Leidsche Rijn Julius Healthcare Centers location Terwijde Utrecht Netherlands; ^5^ Leiden University Medical Center Department of Medical Statistics and Bioinformatics Leiden Netherlands

**Keywords:** COPD, CCQ, health status, eHealth, self-management, integrated disease management, self-efficacy, Web-based platform, primary care, chronically ill, blended care

## Abstract

**Background:**

Worldwide nearly 3 million people die from chronic obstructive pulmonary disease (COPD) every year. Integrated disease management (IDM) improves quality of life for COPD patients and can reduce hospitalization. Self-management of COPD through eHealth is an effective method to improve IDM and clinical outcomes.

**Objectives:**

The objective of this implementation study was to investigate the effect of 3 chronic obstructive pulmonary disease eHealth programs applied in primary care on health status. The e-Vita COPD study compares different levels of integration of Web-based self-management platforms in IDM in 3 primary care settings. Patient health status is examined using the Clinical COPD Questionnaire (CCQ).

**Methods:**

The parallel cohort design includes 3 levels of integration in IDM (groups 1, 2, 3) and randomization of 2 levels of personal assistance for patients (group A, high assistance, group B, low assistance). Interrupted time series (ITS) design was used to collect CCQ data at multiple time points before and after intervention, and multilevel linear regression modeling was used to analyze CCQ data.

**Results:**

Of the 702 invited patients, 215 (30.6%) registered to a platform. Of these, 82 participated in group 1 (high integration IDM), 36 in group 1A (high assistance), and 46 in group 1B (low assistance); 96 participated in group 2 (medium integration IDM), 44 in group 2A (high assistance) and 52 in group 2B (low assistance); also, 37 participated in group 3 (no integration IDM). In the total group, no significant difference was found in change in CCQ trend (*P*=.334) before (–0.47% per month) and after the intervention (–0.084% per month). Also, no significant difference was found in CCQ changes before versus after the intervention between the groups with high versus low personal assistance. In all subgroups, there was no significant change in the CCQ trend before and after the intervention (group 1A, *P*=.237; 1B, *P*=.991; 2A, *P*=.120; 2B, *P*=.166; 3, *P*=.945).

**Conclusions:**

The e-Vita eHealth-supported COPD programs had no beneficial impact on the health status of COPD patients. Also, no differences were found between the patient groups receiving different levels of personal assistance.

**Trial Registration:**

Netherlands Trial Registry NTR4098; http://www.trialregister.nl/trialreg/admin/rctview.asp?TC=4098 (Archived by WebCite at http://www.webcitation.org/6sbM5PayG)

## Introduction

Chronic obstructive pulmonary disease (COPD) is a slowly progressive lung disease and a main cause of morbidity and mortality in high-, middle-, and low-income countries [1]. Worldwide, nearly 3 million people die from COPD every year which, in 2012, was equal to 6% of all deaths globally [2,3].

According to current COPD guidelines, symptoms and airflow obstruction should be regularly monitored to modify treatment and identify complications at an early stage [4,5]. Monitoring should contribute to delaying disease progression and alleviate its manifestations; the most important primary care objective should be to improve quality of life (QoL) [6]. In primary care COPD studies, the mean score on the Clinical COPD Questionnaire (CCQ) reflects a mildly symptomatic COPD [7], and the health status of patients was found to decline over a longer period of time [8].

In the last decade, integrated disease management (IDM) was introduced to improve quality of care. An IDM program consists of different components of care in which different health care providers are cooperating and collaborating to provide efficient and good quality of care; IDM for COPD improves disease-specific QoL and exercise capacity and also reduces hospital admissions and hospital days per person [9].

To improve the quality and efficiency of IDM and reduce health care costs, self-management of COPD patients was introduced and has proven an effective method [10,11,12]. The core components of self-management include education, eliciting personalized goals, psychological coping strategies, improving compliance to treatment, behavioral change, and practical and social support [13,14]. Interventions to support self-management have shown reductions in hospital admissions and fewer sick days because of exacerbations [15,16]. Chronically ill patients who received person-centered care focusing on patient activation and goal setting are better self-managers [17], with self-efficacy as an important factor influencing self-management behaviors [18].

eHealth interventions are effective in stimulating self-management in chronic disease; patients are better able to cope with their illness and adapt their lifestyle, while eHealth support also reduces medical staff consultations [19]. The deployment of eHealth applications facilitates accessibility to health care, enhances patient understanding of their disease, sense of control, and willingness to engage in self-management [20,21]. Although patient attitudes and receptiveness toward eHealth applications are promising in certain groups of age and education [22,23,24], large-scale adoption of eHealth in daily practice still lags behind predictions in comparison with the explosive growth of other digital tools like Facebook and Twitter [25] (also, during recent years, online banking acceptance has increased rapidly worldwide [26]). A major challenge of eHealth in care coordination is to make it beneficial and easy to use for both health care providers and patients [27]. It is important that online self-management support is a fully integrated element of IDM; COPD and asthma patients tended to use an online application on a regular basis when the caregiver was involved, whereas patients on their own used the application only sporadically [28]. For clinicians, the eHealth evidence base needs strengthening, while for primary care practices, a learning process including staff training needs to be instituted [29]. Since advances in eHealth are not clear for patients who have never used it, it is necessary to provide patients with more and better information about the possibilities and potential benefits of eHealth to increase their self-efficacy and provide a feeling of more personal control in daily life [30]. Furthermore, poor user-friendliness of Web-based applications and the lack of push factors (frequent reminders or messages by caregivers) are a common cause of low usage or decline in usage [31].

Despite high expectations and numerous eHealth initiatives, implementation and use of eHealth applications is not yet common practice. Therefore, this e-Vita study investigated the effect of usage of eHealth platforms on the health status of COPD patients treated in primary care. In this paper, we describe 3 eHealth-supported care programs with different components that support the treatment of COPD patients through digital coaching. Two programs were applied as blended care (ie, implemented within usual care to explore the potential for daily health care practice), and one program was applied with the self-management platform as an independent component.

In our e-Vita study, use of the self-management platforms was higher when the platform was an integrated part of IDM and with adequate personal assistance on how to use the platform [32]. We hypothesize that use of the platforms will improve self-management skills and thereby stabilize the health status of COPD patients, with a greater effect with higher usage.

## Methods

### Study Design

Full methodological details of this multilevel parallel cohort design have been reported previously [33]. For our implementation study in a real-life health care setting, we used an interrupted time series (ITS) design in a pragmatic trial in which data were collected at multiple time points before and after implementation to detect whether the intervention had a significantly greater effect than any underlying secular trend [34]. The ITS is performed according to the guidelines of the Effective Practice and Organization of Care Cochrane review group [35].

**Figure 1:**
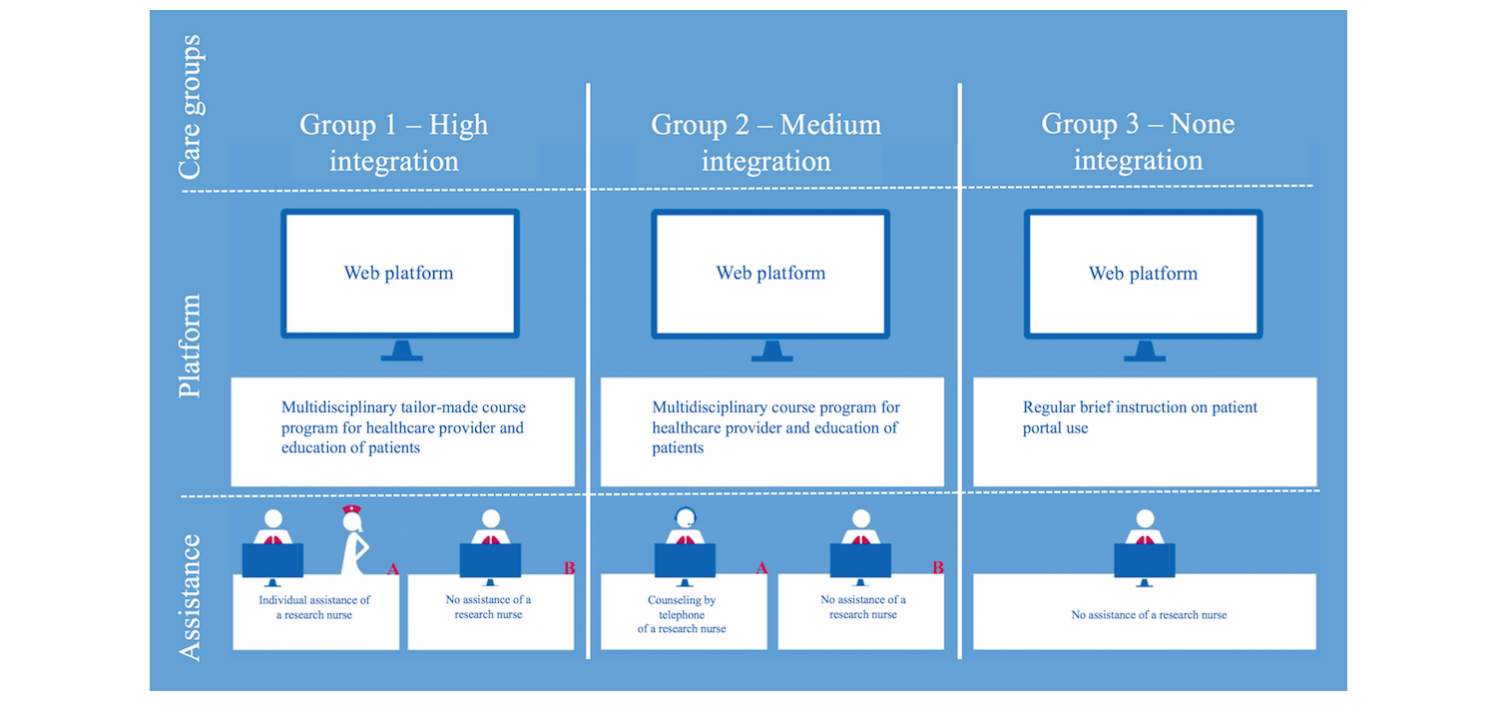
Study design of the e-Vita online platform.

Figure 1 presents an overview of the study design. The study included 3 different care groups in primary care (groups 1 to 3); all patients started using the Web-based platforms (Multimedia Appendices 1 and 2).

In groups 1 and 2, we offered the patients blended care, and in group 3 the self-management platform was offered to the patients as an independent module. In group 1, the online platform was offered as a highly integrated part of the COPD IDM (High) with a tailor-made intensive course program on COPD for health care professionals that contained education on COPD, training about the possibilities of eHealth, and conversational techniques to approach patients in an equal and coaching way. All patients in group 1 started with a personal consultation with the primary care nurse with coaching on the necessity of self-management and explanations of their burden of disease and the eHealth program. Group 2 had a medium level of integration (Medium) with a basic course program for health care professionals on COPD that contained education on COPD and training about the possibilities of eHealth. All patients in group 2 started with a personal consultation with the primary care nurse with coaching and explanation of the self-management program. In groups 1 and 2, the first question the nurse asked was what patients would like to achieve in their daily life when they would have a lower burden of disease. With the platform, the patients could work with a coaching program on their personal goals, actions and health-related QoL [33]. In group 3, the online platform was offered without integration in COPD IDM (None); health care providers and patients received basic instructions on the platform.

Two different levels of assistance for patients were distinguished within groups 1 and 2: one with a high level of personal assistance (A) and the other with a low level of personal assistance (B). Patients were randomly subdivided into 2 groups. In group 1A, high-level support implied home visits to patients by a research nurse who coached and assisted in use of the platform. In group 2A, high-level support implied telephone consultation (3 times during the intervention period, scheduled after 3, 6, and 9 months) between the patient and a research nurse who explained use of the platform. In groups 1B and 2B, low-level support implied that the primary care nurse showed the patient only once how to use the platform. Patients in group 3 who used the online self-management platform had no support from the caregivers or research nurses. Both platforms were provided for the intervention period of 15 months.

### Participants

A total of 3 care groups participated. Patients were eligible when they were diagnosed with COPD according to the Global Initiative for Chronic Obstructive Lung Disease criteria (post-bronchodilator FEV_1_/FVC <0.7) in accordance with the COPD Guidelines of the Dutch College of General Practitioners [36] and when they were being treated for COPD in primary care. Patients were excluded if they were unable to fill in questionnaires, had no access to the Internet, had a terminal illness, were immobile, or were severe substance abusers.

### Recruitment of Patients

We started by recruiting the care groups: managing general practitioners (GPs) in groups 1, 2, and 3 decided to participate because they wanted to contribute to possible health care improvement. Members of the care groups (GPs) volunteered to participate.

Patients were invited to participate by letter via their own GP. When participants of the e-Vita study logged in and used the Web platform at least once, they were defined as users. Patients were defined as lost to follow-up if they did not log on to the platform for at least 12 months after signing informed consent or if they did not complete the digital questionnaires within the intervention period.

### Ethics Approval and Consent to Participate

This study was conducted according to the principles of the Declaration of Helsinki (version 59, 2008) and in accordance with the Medical Research Involving Human Subjects Act. The study was approved by the Medical Ethics Committee of the Medical University Center of Leiden. All subjects provided written informed consent.

### Intervention

The online self-management platforms were created by national experts on chronic disease management guided by interviews with COPD patients about their thoughts and feelings related to living with COPD; the experiences of professional COPD experts were also integrated. The main content of the platform consists of insight into personal health data, self-monitoring of health values (CCQ, Modified Medical Research Council Dyspnea scale [MRC]), education, and a coach for attaining personal goals. The educational and coaching programs were developed by the Lung Alliance of the Netherlands. The online self-management platform e-Vita is an initiative of the Dutch foundation Care Within Reach [37]. The patients received automated online reminders. Details on the online platforms are published elsewhere [33].

### Outcome Measures

The primary outcome is a clinical one, expressed as health status (ie, the CCQ). This questionnaire was designed by Van der Molen [38] and consists of 10 items, each answered on a 7-point Likert scale. The CCQ comprises 3 domains: symptom state (4 items), functional state (4 items), and mental state (2 items). The CCQ total score is calculated as the mean of the sum of all items (minimum 0, maximum 6), with a higher value indicating lower health status. The CCQ is a reliable and valid questionnaire with a Cronbach alpha of 0.89-0.91.

Data collected at baseline included age, gender, education level, and total scores on the CCQ, MRC [39], Generalized Self-Efficacy Scale (GSES) [40], and EuroQol 5-dimension questionnaire (EQ5D) [41]. Education was self-reported using 8 response categories ranging from no formal education to graduate or professional level and converted into 3 levels (low, medium, high). In the main analyses, personal assistance for the participants (high assistance vs low assistance) and integration in IDM (integrated vs not integrated) were used as determinants.

### Data Collection

**Figure 2:**
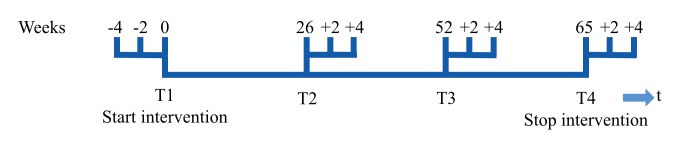
Interrupted time series measurements of the Clinical COPD Questionnaire per interval.

Data were extracted from the log files of the self-management platforms. Figure 2 shows the measurement schedule of the CCQ. During the 15-month intervention period, there were 4 measurement periods with 3 CCQ questionnaires at each period (3 data points before intervention and 9 data points after intervention) in order to apply ITS analysis.

### Sample Size Calculation

Generally, the health status of patients with COPD decreases over time. Studies on IDM in COPD in primary care show that a general increase in CCQ of 0.5 (SD 0.75) can be expected over a 1-year period [42,43]. In this study, we offered patients a Web platform in addition to their regular IDM program. Therefore, we hypothesized that the regular increase in CCQ (0.5 points per year) would change to stabilization of health status as compared to literature [44].

To measure differences in health status (>0.5 CCQ points) at 80% power (SD 0.75 and α=0.05), 37 patients needed to be included. Based on an estimated 20% drop-out during the study period, 45 (37/0.80) patients needed to be included. As we used 2 different implementation methods (with and without personal assistance) in 2 of the care groups, 2×45=90 patients needed to be included in those groups. In the third care group, because only 1 implementation method was used, a total of 45 patients were required to achieve sufficient statistical power.

### Statistical Methods

Categorical baseline characteristics were reported as numbers and percentages, normally distributed continuous variables as means with standard deviations (SD), and nonnormally distributed variables as medians with interquartile ranges (IQRs). Characteristics between the 3 groups were examined using chi-square tests and Kruskal-Wallis tests.

ITS analyses were used to study time trends before and after intervention. Due to the correlation between the repeated measurements within a patient, we used multilevel linear regression modeling (mixed models) to analyze CCQ data in the total group as well as in the groups with high and low levels of assistance and in the 5 subgroups.

The analyses allowed us to quantify the effect of the intervention on CCQ versus the observed preintervention period. Estimates for regression coefficients corresponding to 2 standardized effect sizes were obtained: a direct change in the level of the CCQ (also called step change or jump) and a change in trend of the CCQ before and after the intervention [34].

Included in the model for the total group and 5 subgroups as fixed effects were time, treatment, and the interaction between time and treatment; the model comparing the groups with high and low assistance additionally contained the assistance group factor and the interaction of this factor. All models included a random intercept per patient. When there was a substantial improvement in the Akaike Information Criterion (used to assess the model fit score), an additional random slope (time) was used.

Because of a nonnormal distribution of the CCQ data, the log of the CCQ data was used as outcome in the multilevel linear regression models. The analysis model did not test or correct for seasonality; although seasonality influences exacerbations of COPD, it has no effect on the CCQ [45,46]. We visually assessed normality of the residuals to evaluate the validity of the assumptions of the mixed models analysis.

## Results

### Inclusion

**Figure 3:**
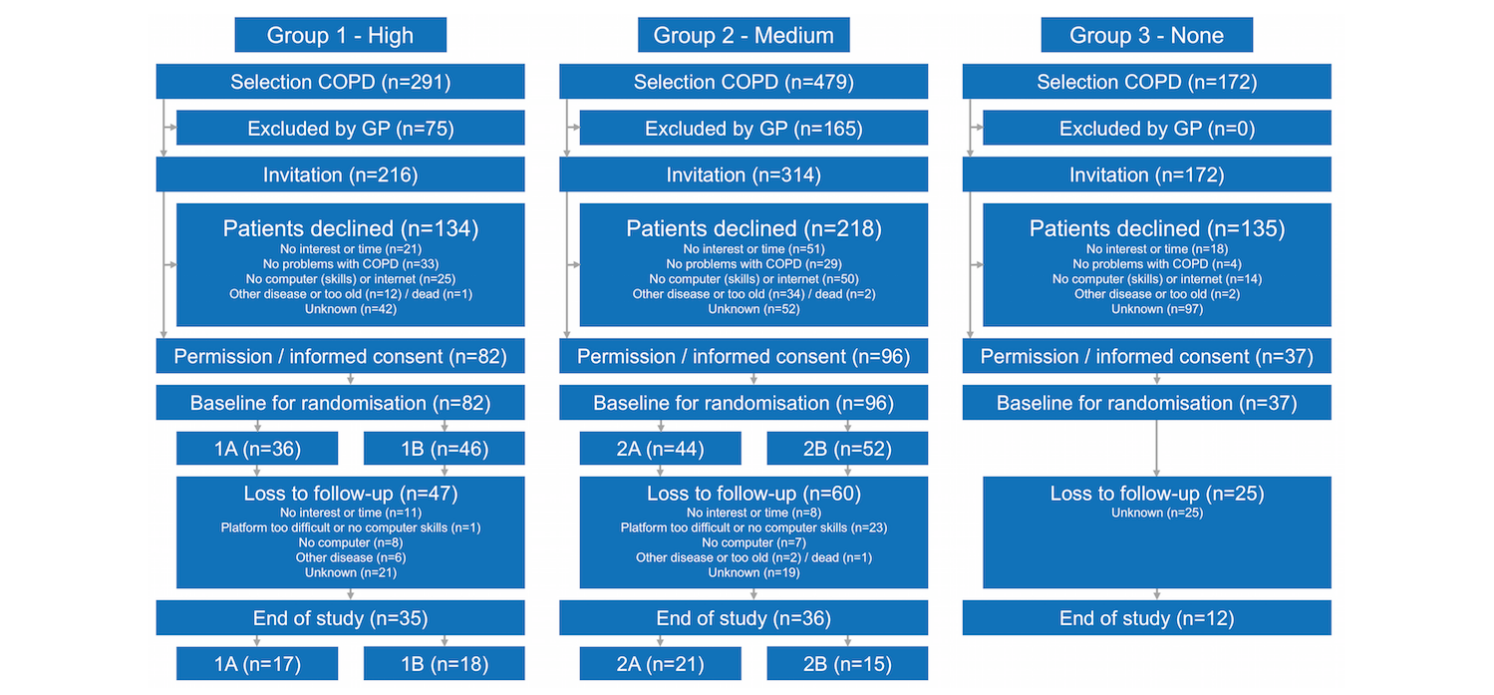
Flowchart of the e-Vita chronic obstructive pulmonary disease study.

A total of 942 COPD patients were selected from 3 care groups (Figure 3). The GPs of these care groups excluded 240 COPD patients from participation due to other diseases, treatment in hospital, or incompetency to participate in the program. Finally, 702 COPD patients were invited, of whom 215 (30.6%) agreed and provided informed consent.

The number of patients lost to follow-up (no log on to the platform after signing informed consent or not completing the entire intervention period) was 132; results of the nonparticipation analysis are reported elsewhere [33]. Figure 3 shows the reasons for loss to follow-up in groups 1 and 2; patients in group 3 were not asked for their reasons.

### Baseline Characteristics of Patients With Chronic Obstructive Pulmonary Disease

Table 1 presents the baseline demographic and clinical characteristics of the included COPD population (median age 66.6 years; 52.1% male). These patients had mildly symptomatic COPD which was reflected by a median MRC scale of 1.0 and a median CCQ of 1.2. Out of 215 participants, 89 (41.4%) filled in the online questionnaire for education level; most participants had a middle education level, reflected by a 4 or 5 on a scale of 1 to 7 (38/89, 42.7%). The median GSES was 3.3, and the median EQ-5D was 0.86. The characteristics age (χ^2^_2_=5.4, *P*=.07), education level (χ^2^_4_=2.2, *P*=.70), GSES (χ^2^_2_=1.7, *P*=.42), and EQ-5D (χ^2^_2_=2.4, *P*=.28) were similar in the 3 groups. There was a difference in the characteristic gender (χ^2^_2_=6.8, *P*=.03), with more male patients in group 1, and a difference in CCQ (χ^2^_2_=6.5, *P*=.04) and MRC scale (χ^2^_2_=11.3, *P*=.003), with a higher CCQ and MRC scale in group 2.

**Table 1:** Baseline characteristics of patients with chronic obstructive pulmonary disease in the e-Vita study.

Participants		Group 1 (high)		Group 2 (medium)		Group 3 (none)	Total
Assistance		High n=36	Low n=46	High n=44	Low n=52	n=37	n=215
Age, years, (IQR^a^)		66.3 (61.0-79.2)	65.6 (61.3-73.4)	68.7 (64.0-78.3)	66.8 (60.3-75.1)	64.1 (61.5-69.2)	66.6 (61.4-74.7)
Males, n (%)		19 (52.8)	32 (69.6)	17 (38.6)	24 (46.2)	20 (54.1)	112 (52.1)
**Education level, n (%)**							
	Low	4 (28.6)	8 (38.1)	5 (22.7)	8 (42.1)	7 (53.8)	32 (36.0)
	Medium	7 (50.0)	8 (38.1)	11 (50.0)	8 (42.1)	4 (30.8)	38 (42.7)
	High	3 (21.4)	5 (23.8)	6 (27.3)	3 (15.8)	2 (15.8)	19 (21.3)
**Questionnaire, median (IQR)**						
	CCQ^b^	1.0 (0.6-1.9)	1.2 (0.8-1.6)	1.3 (0.9-2.1)	1.4 (1.1-2.1)	1.3 (0.6-1.8)	1.2 (0.8-1.9)
	MRC^c^	1.0 (1.0-3.0)	1.0 (1.0-2.0)	2.0 (1.0-3.0)	2.0 (1.0-2.0)	1.0 (1.0-1.0)	1.0 (1.0-2.0)
	GSES^d^	3.4 (3.1-3.7)	3.3 (3.0-3.8)	3.3 (2.8-3.5)	3.3 (3.1-3.7)	3.4 (3.3-3.7)	3.3 (3.0-3.7)
	EQ-5D^e^	0.85 (0.7-1.0)	0.89 (0.81-1.0)	0.85 (0.72-1.0)	0.84 (0.71-1.0)	0.9 (0.84-1.0)	0.86 (0.78-1.0)

^a^IQR: interquartile range.

^b^CCQ: Clinical COPD Questionnaire.

^c^MRC: Modified Medical Research Council Dyspnea score.

^d^GSES: Generalized Self-Efficacy Scale.

^e^EQ-5D: EuroQoL 5-Dimension Questionnaire.

### Health Status Changes

**Figure 4:**
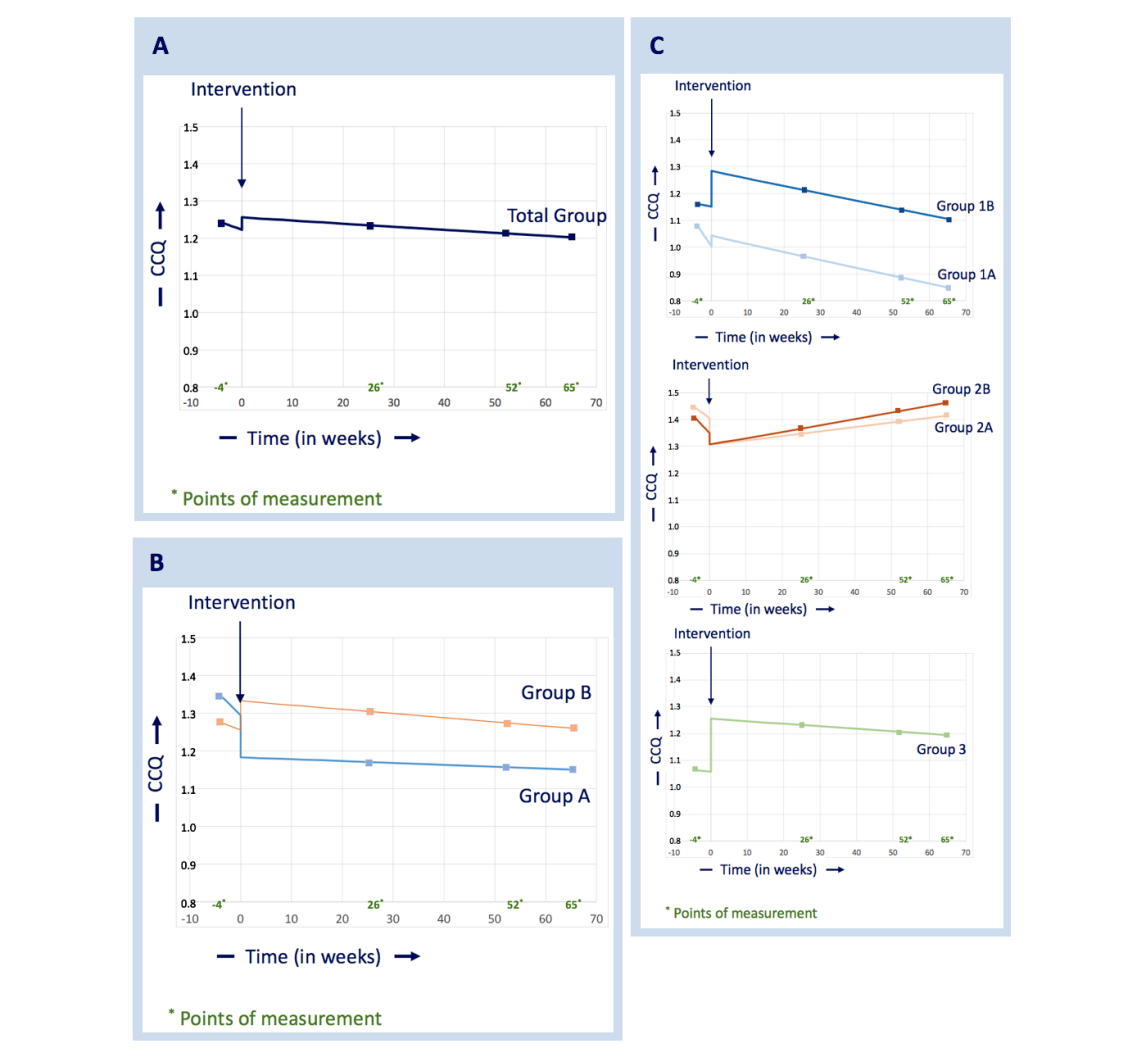
(A) Total group analysis of health status, (B) analysis of health status in groups receiving high and low levels of assistance, and (C) subgroup analyses of health status.

Figure 4 A shows the effect of the intervention on CCQ in the total patient group. The decrease before the intervention was 0.5% per month and after the intervention 0.08% per month; this difference was not significant (*P*=.334). The estimated direct change in the level of the CCQ slopes at the moment of the intervention (jump) was –0.015 (*P*=.421) implying that the CCQ trend was 1.5% lower before the intervention.

Figure 4 B shows the effect of the intervention on CCQ in the groups with a high level of personal assistance (A) and a low level of personal assistance (B). In group A, the preintervention decrease was 0.8% per month and the decrease after the intervention was 0.05% per month; in group B, the preintervention decrease was 1.8% per month and the decrease after the intervention was 0.1% per month. No significant difference was found in CCQ changes going from preintervention to postintervention between groups A and B (*P*=.429). The direct change in the level of the CCQ slopes at the moment of the intervention (jump) was 0.017 in group A and –0.033 in group B (implying that the CCQ trend was 1.7% higher before the intervention in group A and 3.3% lower in group B). There was no significant difference in the jumps (*P*=.207).

Figure 4 C shows the effect of the intervention on the CCQ in the 5 subgroups; no significant difference was found in the slope of the CCQ before and after the intervention (1A, *P*=.237; 1B, *P*=.991; 2A, *P*=.120; 2B, *P*=.166; 3, *P*=.945). The direct changes in the level of the CCQ slopes before and after the intervention (jump) were –0.0196 in group 1A, –0.0582 in group 1B, 0.0426 in group 2A, 0.0184 in group 2B, and –0.0874 in group 3 and were not significant.

## Discussion

### Principal Findings

This study investigated the effect of use of eHealth platforms on the health status of COPD patients in primary care. No changes in health status were found before and after introduction of the eHealth-supported COPD programs, and no differences were found between care groups with a high versus a low level of personal assistance.

It is essential to carefully review the design strategies for integrating eHealth applications within disease management [47] by means of a thorough evaluation and analysis of the results. A recent study analyzed the effect of wearable devices that monitored and provided feedback on physical activity among young adults with obesity; providing a group with this device resulted in less weight loss over 24 months [48]. In a trial of elderly persons with a high risk for prehospitalization, telemonitoring was offered for monitoring and reporting symptoms but did not result in lower hospitalizations or emergency room visits (although mortality was higher in this telemonitoring group) [49]. High expectations of eHealth should be preceded by evaluations in pragmatic studies on implementation [50].

In our study, changes in health status CCQ were not within the range of a minimal clinically important difference [51]. QoL and health status are determined by a significant number of factors [46]. We expect that eHealth interventions will be effective in stimulating self-management and stabilizing health status in COPD patients when these patients use the platforms for a longer period of time. In earlier studies, a worsening in health status was found for primary care patients over a longer period of time [8]; this finding was not confirmed in our e-Vita study. In Dutch primary care, the standards of IDM are high, with a wide variety in the implementation of interventions [7]; this might explain the absence of a worsening of health status before the intervention in our study. The introduction and integration of eHealth within IDM will not make a significant difference in the short term due to the high standard of IDM.

In our study, the median baseline CCQ score of 1.2 was low compared with scores in other primary care COPD studies, which reflects mildly symptomatic COPD [7]. This limits the room for stabilization or improvement in our primary outcome (ie, ceiling effect).

In a patient population with more severe COPD (patient-data meta-analysis from 2016), self-management interventions improved health-related QoL at 12 months but not at 6 months [52]; this confirms our observation that long-term use of platforms is necessary for an effect on health status. In our research, the platforms are probably not sufficiently customized to the wishes or needs of COPD patients to provide sufficient motivation to use the platform on a regular basis for a longer period; in our e-Vita study, a significant number of users stopped using the platforms (attrition) [32].

The change in level of CCQ (positive/negative) at the start of the intervention might be explained by the participant rise in consciousness regarding their health status, thereby completing the questionnaire more critically after explanation from a health care professional. Similar to our study, in a randomized controlled trial (RCT) with asthma patients, the QoL was enhanced over the first 3 months after starting to use a self-management portal [53].

The effect of eHealth cannot easily be evaluated in a classical RCT; integrating eHealth in IDM is a complex intervention in a multidisciplinary care process. Pragmatic trials frequently include complex interventions and often involve the skills and experience of health care professionals [54] and are, therefore, more suitable for eHealth studies.

EHealth technologies for chronic conditions can be used to enhance self-management and revise the chronic care model; patients who actively participate in their care achieve valuable and sustained improvement in well-being [55,56]. Findings in many eHealth studies suggest that the use of a personal health record or self-management platform can promote an informed or activated patient and augment the chronic care model for self-management support and productive interactions even though a direct dosage-effect relationship (usually analyzed in a classical RCT) is not common in eHealth studies [57]. Also in our e-Vita study, interpretation of the results on use and health status cannot be made in a direct dosage-effect relationship. Use of the self-management platform was higher when the platform was an integrated part of IDM, with trained caregivers encouraging patients to use the platform and with personal assistance about how to use the platform, but without a significant change in health status. Based on current literature and the e-Vita COPD study, we conclude that eHealth-supported self-management integrated into usual care can help patients with COPD manage their disease better.

Further studies based on this study and current literature are needed to establish the mechanisms most likely to ensure the successful development and implementation of Web-based self-management interventions, including considerations about how the intervention is integrated in IDM and how it enhances the patient’s self-management behavior to stimulate long-term use of self-management platforms with a stabilizing effect on health status [11,58].

### Strengths and Limitations

This e-Vita COPD study has several strengths. To our knowledge, it is the first to combine different study designs thereby enabling simultaneous investigation of the effect of eHealth and the effects of different organizational implementation methods on health status. Randomization was performed for the level of assistance provided to patients, allowing comparison of patient groups with high and low levels of assistance. Because the care groups 1, 2, and 3 were not randomized, no analysis of the differences between these groups can be made.

An advantage of the ITS design is that it detects changes that are delayed or intermittent and can determine whether the change is permanent or temporary. The design, including the 3 datapoints of CCQ before the intervention, also allows evaluation of variables which are changing by comparing slopes of trend lines before and after the intervention.

This study also has some limitations. Development of the platforms was relatively difficult due to lack of experience in this field. Also, decisions made during the design phase were beyond the influence of our group but affected the usability of the platforms. Self-management skills imply behavioral changes which require some time, whereas the present study period was restricted to 15 months. Furthermore, patients in a primary care setting have a low burden of disease (in this study, a median score on the CCQ of 1.2) and motivation to use the platform might be negatively influenced by this fact. In respiratory medicine there is a lack of research on patients with mild-to-moderate COPD despite that over 80% of COPD patients suffer from this stage of disease and are often treated in primary care [59]. Other projects among care groups (eg, patient education, start different IDMs) might influence the speed and thoroughness of the implementation of our platforms.

This study also has limitations typically associated with eHealth trials. For example, as GPs and patients were free to volunteer, bias might have occurred in our study groups. Users were self-selected and were, presumably, motivated to use the Web-based platform as would be expected in a real-life setting. Also, the patients selected to be invited by the GPs might differ from other patient groups. Furthermore, GPs excluded 25.5% of the COPD patients from this study. Of the 702 eligible patients, 30.6% were willing to participate and provided informed consent, and 61.4% of the participants dropped out during follow-up. Even though the nonparticipants did not differ in age or gender from the participants [32], caution is required when generalizing these results to general practice.

Like most Internet outcome studies, there were 2 types of attrition in our study; attrition from the intervention itself (lack of site utilization) and attrition from the follow-up assessments. This law of attrition (the phenomenon of participants stopping usage) is a common finding in eHealth evaluations and one of the fundamental and methodological challenges in the evaluation of eHealth applications [60]. To prevent both types of attrition, email reminders were sent by the platform to fill in the questionnaires. All users received urgent and repeated requests to fill in questionnaires by email and by telephone. The attrition curve was analyzed earlier and depicts the push factors that are required to remind participants to use the platform [32]. The loss to follow-up is high with a risk of biased results due to user bias; therefore, these results are only applicable for users of eHealth.

The study aimed to be inclusive rather than exclusive to achieve higher external validity. Patients were excluded if they were unable to fill in questionnaires, had no access to the Internet, had a terminal illness, were immobile, or were severe substance abusers. During the inclusion of patients, we found that patients did not want to start the study for several different reasons: no computer skills, old age, no problems with COPD, and other reasons. These reasons are typical for eHealth research in a primary care setting with a low burden of disease; participants were self-selected and were, presumably, motivated to use the Web-based platform as would be expected in a real-life setting. Therefore, the results of the study are not generalizable to all COPD patients but to those who are willing, motivated, and able to use eHealth. Nevertheless, we believe that this study is inclusive rather than exclusive, since there are almost no limitations for participation for this group of motivated patients.

However, the practical applicability of our results for other primary care groups is positive (ie, the study provides practical insight into successful implementation of patient platforms). Nevertheless, primary care organizations should take into account the different aspects of the organization of blended care and quality of implementation.

Although an RCT provides the most reliable evidence on the effectiveness of interventions, this was not feasible for our implementation study in a real-life health care setting with 3 different care groups. After randomization in groups 1 and 2, more patients were assigned to the groups with a low level of personal assistance (group 1B and 2B). After simple randomization, some discrepancy between the numbers in the comparison groups would be expected [61]. Such unpredictability reflects the essence of randomness. Moreover, the baseline characteristics did not differ significantly between groups with high and low assistance. Therefore, we expect no significant influence on the results.

To measure a significant difference in health status, 45 patients were needed in each subgroup; although these numbers were not met within each subgroup, analysis on the combined groups should be sufficiently powered to detect relevant differences. In addition, the number of data points collected before the intervention has a substantial impact on the strength of an ITS design. It is necessary to collect enough data points to be convinced that a stable estimate of the underlying secular trend has been obtained [62]. In our study, the 3 data points before the intervention represent a minimum number of data and may have influenced the effective power of our study.

### Conclusion

There is growing interest in the potential of Web-based self-management platforms to deliver more individually tailored self-management support integrated into the everyday lives of COPD patients to improve their quality of life. In this study, the e-Vita eHealth-supported COPD programs had no significant impact on the health status of COPD patients, health status showed no significant change before or after the introduction of the eHealth-supported programs, and no differences were found between the patient groups receiving different levels of personal assistance.

## Appendix

Multimedia Appendix 1 Homepage e-Vita.

Multimedia Appendix 2 Homepage Zorgdraad.

Multimedia Appendix 3 SQUIRE checklist.

## Abbreviations

CCQ: Clinical COPD Questionnaire
COPD: chronic obstructive pulmonary disease
EQ-5D: EuroQol 5-dimension questionnaire
FEV: forced expiratory volume
FVC: forced vital capacity
GP: general practitioner
GSES: Generalized Self-Efficacy Scale
IDM: integrated disease management
IQR: interquartile range
ITS: interrupted time series
MRC: Modified Medical Research Council Dyspnea scale
RCT: randomized controlled trial
QoL: quality of life
