# Supplementary figures and images for: The Effect of Integration of Self-Management Web Platforms on Health Status in Chronic Obstructive Pulmonary Disease Management in Primary Care (e-Vita Study): Interrupted Time Series Design
Source: J Med Internet Res. 2017 Aug 16;19(8):e291. doi: 10.2196/jmir.8262 (PMC5577456; doi:10.2196/jmir.8262)

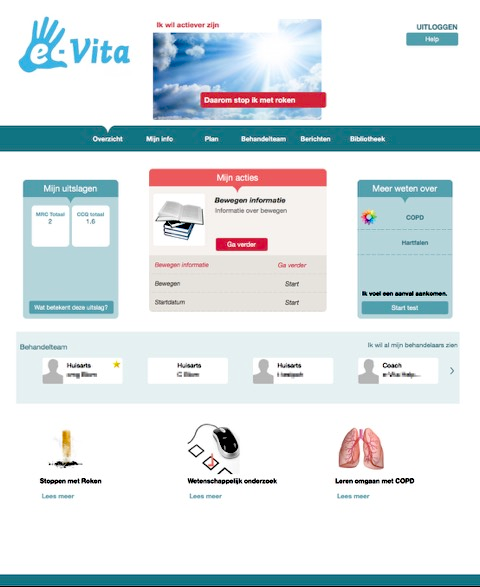

Supplement: Multimedia Appendix 1 [file jmir_v19i8e291_app1.png]

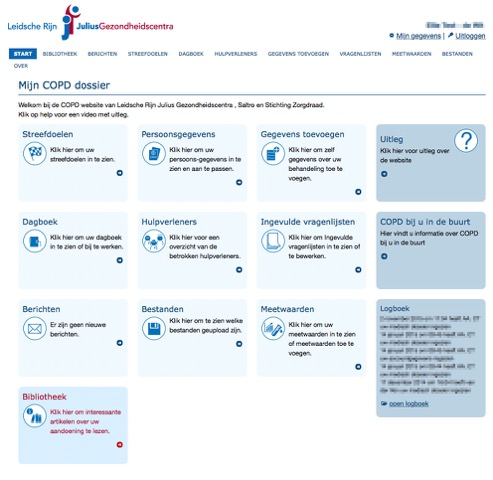

Supplement: Multimedia Appendix 2 [file jmir_v19i8e291_app2.png]
