# Supplementary material for: The Effect of Integration of Self-Management Web Platforms on Health Status in Chronic Obstructive Pulmonary Disease Management in Primary Care (e-Vita Study): Interrupted Time Series Design
Source: J Med Internet Res. 2017 Aug 16;19(8):e291. doi: 10.2196/jmir.8262 (PMC5577456; doi:10.2196/jmir.8262)
Supplement: Multimedia Appendix 3 [file jmir_v19i8e291_app3.pdf]

Research and reporting methodology
Revised **Standards for QUality Improvement Reporting Excellence**
(**SQUIRE 2.0**) publication guidelines

**Text section and item name**	**Page**
	**info is located**
**Title and abstract**	
1. **Title**	
Indicate that the manuscript concerns an initiative to improve healthcare (broadly defined to include the quality, safety, effectiveness, patient-centredness, timeliness, cost, efficiency and equity of healthcare).	Page 1
2. **Abstract**	
a. Provide adequate information to aid in searching and indexing.	Page 2 (Abstract/Keywords/ Article summary)
b. Summarise all key information from various sections of the text using the abstract format of the intended publication or a structured summary such as: background, local problem, methods, interventions, results, conclusions.	Page 2 (Abstract/Article summary/What this paper adds)
**Introduction: Why did you start?**	
3. **Problem description** - Nature and significance of the local problem.	Page 4 (Introduction)
4. **Available knowledge** - Summary of what is currently known about the problem, including relevant previous studies.	Page 4 (Introduction)
5. **Rationale** - Informal or formal frameworks, models, concepts and/or theories used to explain the problem, any reasons or assumptions that were used to develop the intervention(s) and reasons why the intervention(s) was expected to work	Page 4/5 (Introduction)
6. **Specific aims** - Purpose of the project and of this report.	Page 5 (Introduction)
**Methods: What did you do?**	
7. **Context** - Contextual elements considered important at the outset of introducing the intervention(s).	Page 5 (Study design)
8. **Intervention(s)**	
a. Description of the intervention(s) in sufficient detail that others could reproduce it.	Page 6/7 (Intervention)
b. Specifics of the team involved in the work.	Page 6/7 (Intervention)
9. **Study of the intervention(s)**	
a. Approach chosen for assessing the impact of the intervention(s).	Page 5/6 (Study design)
b. Approach used to establish whether the observed outcomes were due to the intervention(s).	Page 7/8 (Statistical methods)
10. **Measures**	
a. Measures chosen for studying processes and outcomes of the intervention(s), including rationale for choosing them, their operational definitions and their validity and reliability.	Page 7 (Outcome measures)

b. Description of the approach to the ongoing assessment of contextual elements that contributed to the success, failure, efficiency and cost.	Page 7 (Outcome measures)
c. Methods employed for assessing completeness and accuracy of data.	Page 7/8 (Statistical methods)
11. **Analysis**	
a. Qualitative and quantitative methods used to draw inferences from the data.	Page 7/8 (Statistical methods)
b. Methods for understanding variation within the data, including the effects of time as a variable.	Page 7/8 (Statistical methods)
12. **Ethical considerations** - Ethical aspects of implementing and studying the intervention(s) and how they were addressed, including, but not limited to, formal ethics review and potential conflict(s) of interest.	Page 14 (Ethics approval and consent to participate / Competing interests)
**Results: What did you find?**	
13. **Results**	
a. Initial steps of the intervention(s) and their evolution over time (eg, time-line diagram, flow chart or table), including modifications made to the intervention during the project.	Page 8/9 (Results)
b. Details of the process measures and outcomes.	Page 9 (Baseline characteristics of COPD patients/ Health status before/after intervention in the total group, in groups with/without personal assistance, and in the subgroups)
c. Contextual elements that interacted with the intervention(s).	Page 9 (Baseline characteristics of COPD patients/ Health status before/after intervention in the total group, in groups with/without personal assistance, and in the subgroups)
d. Observed associations between outcomes, interventions and relevant contextual elements.	Page 9 (Health status before/after intervention in the total group, in groups with/without personal assistance,

	and in the subgroups)
e. Unintended consequences such as unexpected benefits, problems, failures or costs associated with the intervention(s).	Page 10 (Strengths and limitations)
f. Details about missing data.	Page 8/9 (Results)
**Discussion: What does it mean?**	
14. **Summary**	
a. Key findings, including relevance to the rationale and specific aims.	Page 10 (Discussion)
b. Particular strengths of the project.	Page 10 (Strengths and limitations)
15. **Interpretation**	
a. Nature of the association between the intervention(s) and the outcomes.	Page 11/12 (Interpretation of findings)
b. Comparison of results with findings from other publications.	Page 11/12 (Interpretation of findings)
c. Impact of the project on people and systems.	Page 11/12 (Interpretation of findings)
d. Reasons for any differences between observed and anticipated outcomes, including the influence of context.	Page 11/12 (Interpretation of findings)
e. Costs and strategic trade-offs, including opportunity costs.	N/A; this manuscript does not look at costs.
16. **Limitations**	
a. Limits to the generalisability of the work.	Page 10/11 (Strengths and limitations)
b. Factors that might have limited internal validity such as confounding, bias or imprecision in the design, methods, measurement or analysis.	Page 10/11 (Strengths and limitations)
c. Efforts made to minimise and adjust for limitations.	Page 10/11 (Strengths and limitations)
**Conclusions**	
a. Usefulness of the work.	Page 11/12 (Interpretation of findings)
b. Sustainability.	Page 11/12 (Interpretation of findings)
c. Potential for spread to other contexts.	Page 11/12 (Interpretation of

	findings)
d. Implications for practice and for further study in the field.	Page 11/12 (Interpretation of findings)
e. Suggested next steps.	Page 11/12 (Interpretation of findings)
**Other information**	
18. **Funding** - Sources of funding that supported this work. Role, if any, of the funding organisation in the design, implementation, interpretation and reporting.	Page 14 (Funding)

Ogrinc G, et al. BMJ Qual Saf 2015;0:1–7. doi:10.1136/bmjqs-2015-004411
